# Effect of attentional bias modification on social media addiction: a visual dot-probe task

**DOI:** 10.3389/fpsyg.2026.1792101

**Published:** 2026-03-20

**Authors:** Jin Zhao, Jialing Yu, Zhishen Shi, Minghui Li, Xinyu Zhou, Zhanfang Liu, Lei Hao

**Affiliations:** 1 School of Art and Media, Guangzhou Vocational University of Science and Technology, Guangzhou, China; 2 School of Preschool Education, Xiangnan Preschool Education College, Chenzhou, China; 3 Department of Psychology, The Fourth People’s Hospital of Liaocheng, Liaocheng, China

**Keywords:** attentional bias modification, college student, intervention, social media addiction, visual dot-probe task

## Abstract

**Introduction:**

Social media addiction (SMA) negatively affects individuals’ physical wellbeing and mental health. The attentional bias (AB) toward stimuli associated with social media may significantly contribute to the development of SMA. This study examines the use of an attentional bias modification (ABM) program as an intervention and investigates both the short-term and long-term effects of such intervention methods.

**Methods:**

A total of 44 college students exhibiting SMA tendencies were divided into a training group, which received ABM training, and a control group, which received non-biased attention training. Changes in AB and SMA severity were compared between the two groups at four distinct time points: prior to training (Time 1), immediately following training (Time 2), 2 weeks post-training (Time 3), and 4 weeks post-training (Time 4), to assess the effects of ABM training on SMA.

**Results:**

Following ABM training, participants in the training group exhibited a shift away from social media relevant AB and toward attentional avoidance at Time 2, with a concomitant reduction in the SMA severity. The intervention effects were found to be sustained at Time 3 and Time 4. By contrast, the control group demonstrated no significant changes in either their AB toward social media relevant information or the SMA severity at any of the four time points.

**Discussion:**

This study thus substantiates the efficacy of ABM training in mitigating SMA, providing a new method for intervention and treatment.

## Introduction

In contemporary society, social media platforms such as Facebook, WeChat, and TikTok have become deeply embedded in everyday life, but this makes it challenging for many people to conduct their daily routines without them. Social media empowers users to transcend geographical and spatial constraints to access global sources of information, connect with others, and acquire extensive knowledge. Nonetheless, overuse and excessive engagement with social media can result in social media addiction (SMA).

### Social media addiction: a growing public health concern in the digital age

Social media addiction is a form of behavioral addiction characterized by excessive, compulsive, and uncontrollable use of social media platforms, leading to significant negative consequences across multiple life domains (Kuss and Griffiths, 2011). A significant amount of research has shown that SMA is similar to other types of addictive behaviors, like gambling and substance abuse, and manifests comparable addictive symptoms (Eichenberg et al., 2024; Nikolinakou et al., 2024; Sun and Zhang, 2021). SMA has emerged as a substantial concern in the digital age (Pellegrino et al., 2022). Pieces of evidences have indicated that SMA may result in a variety of adverse outcomes among adolescents and young adults, including diminished academic performance, reduced subjective well-being, and association with eating behaviors both directly and also indirectly through deteriorating body image (Mohsenpour et al., 2023; Perez-Lozano and Espinosa, 2024; Zhao et al., 2022a).

Given these potential adverse consequences of SMA, public health practitioners, clinical researchers, and psychologists have gradually begun focusing their attention on identifying the most effective interventions for preventing and alleviating such addictive behaviors (Brevers and Turel, 2019; Chen et al., 2022; Dogan et al., 2019; Hou et al., 2019). For instance, Hou et al. (2019) used cognitive behavioral therapy over a one-week period to address SMA. They found that SMA severity decreased in the experimental group, and that their mental health, self-esteem, and sleep quality showed improvement after therapy as well. By contrast, the control group did not exhibit any notable changes in comparison to the baseline measurements. Although cognitive behavioral therapy can effectively mitigate the risks of behavioral addiction in the short term, roughly half of the individuals given such treatment will experience a relapse within the next year (Cutler and Fishbain, 2005). One possible reason for this is that cognitive behavioral therapy primarily focuses on explicit decision-making processes rather than the key factors that sustain addictive behaviors.

### Attentional bias and addiction

Attentional bias (AB) is a form of cognitive bias in which a person tends to focus more attention on particular types of stimuli (MacLeod et al., 2002). Some studies suggest that AB toward social media-related information is a key factor in the maintenance and development of SMA. For example, Nikolaidou et al. (2019) found a significant positive correlation linking AB toward social media-related stimuli and SMA levels. Similarly, Zhao et al. (2022b), using the dot-probe task, found that individuals with SMA tendencies respond faster to social media-related information than to neutral information. The connection between AB in response to social media relevant cues and SMA can be explained in terms of the incentive-sensitization theory (Robinson, 1993). This theory posits that individuals assign motivational value to social media-related information through a process of classical conditioning (Pavlov, 2010). Specifically, social media-related information becomes salient due to its continued association with rewarding experiences (e.g., experiencing pleasure or escaping negative emotions) (Robinson and Berridge, 2002, 2008). Social media relevant cues will then automatically capture the attentional resources of individuals with a tendency toward SMA, which will in turn drive increased use of social media. Kakoschke et al. (2014) found that cues associated with addiction can automatically attract the attention of individuals with addictive tendencies, bypassing conscious awareness. Some scholars have proposed that AB, which is characterized by plasticity, can be effectively modified through training (Amir et al., 2023; Nuijs et al., 2025). This procedure is known as attentional bias modification (ABM) training.

### Applications of attentional bias modification training in addiction treatment

ABM is a novel intervention that is specifically designed to target and modify cognitive processes. The most frequently used version of ABM is the modified dot-probe task, which is an extension of the classical dot-probe task (Mogoase et al., 2014). In the classic dot-probe task, a set of stimuli (e.g., images related to alcohol and pictures of neutral everyday items) is displayed simultaneously on opposite sides of the computer screen (MacLeod et al., 1986). A probe stimulus is then presented instantly after this stimulus pair disappears. The probe target randomly appears in the position previously occupied by one of the two stimuli, and participants are required to identify its location on the screen (e.g., left or right) (Mogg and Bradley, 1999). Although the classic dot-probe task is designed to measure AB, the ABM paradigm is designed to manipulate it, in accordance with the specific training objectives (MacLeod et al., 2002). When the training objective is to increase AB toward a specific stimulus, the probe target is typically presented more frequently, or even exclusively, at the location of that stimulus. Conversely, when the goal is to decrease AB toward a stimulus, the probe target is presented more frequently at the location of the alternative stimulus.

Attentional bias modification training has been increasingly applied in addictive behaviors and related disorders, yet overall findings remain mixed across studies and domains. Several meta-analyses and systematic reviews have indicated that ABM tends to yield small-to-moderate effects on AB indices, which represent cognitive-level outcomes. However, effects on clinical or behavioral symptoms, such as craving, addictive behavior, or symptom severity, are often small or nonsignificant (Heitmann et al., 2018; Kruijt et al., 2019; Heitmann et al., 2021). In other words, ABM often successfully modifies attentional allocation, but such cognitive changes do not always translate into meaningful improvements in real-world addictive behaviors.

Although the overall effect of ABM in the intervention of addictive behaviors is not yet significant, its potential value still attracts extensive attention from the academic community, and a large number of studies continue to explore its mechanism of action and optimization paths. For example, Smith et al. (2020) provided empirical evidence supporting the effectiveness of ABM training in reducing addiction-related AB. By administering ABM training targeted at food cues among overweight women, their research demonstrated that ABM could effectively redirect attention away from reward-related stimuli. Notably, only the active training group exhibited reduced AB toward food cues and their food intake was observed to decrease, whereas the placebo and control groups did not show similar changes. This between-group difference highlights the specific effect of ABM in modifying attentional allocation, rather than non-specific practice or test–retest effects, thus reinforcing the potential of ABM as a targeted intervention for addictive behaviors.

However, another study has reported null or negligible effects on clinical outcomes. In a study of nicotine dependence, Robinson et al. (2022) reported that although ABM successfully reduced AB toward smoking-related cues, but it did not translate to reduced craving or improved smoking cessation outcomes. These findings revealed that reduced AB toward addiction-related cues alone may not be sufficient to modify actual addictive behavior, highlighting a potential gap between cognitive-level changes and clinical outcomes. This inconsistency across studies further indicates that the efficacy of ABM may depend on factors such as target population, training duration, and the type of addictive behavior, emphasizing the need for a more nuanced theoretical understanding of when and how ABM contributes to the intervention of addiction.

According to incentive-sensitization theory, addictive behavior could potentially be reduced by modifying the underlying cognitive processes, and particularly by modifying the AB toward addiction-related information (Robinson, 1993). Therefore, we propose that reducing AB toward social media relevant cues may be a successful strategy for reducing SMA. The present study is designed to examine the effect of ABM on SMA, particularly in terms of its impact on AB and SMA severity. The hypotheses of this study are as follows:

*H1:* Following ABM training, the training group will demonstrate a reduction in AB toward social media relevant cues.

*H2:* After undergoing ABM training, the training group will exhibit a decrease in SMA severity.

## Materials and methods

### Participants

Calculations using G*Power, with the parameters *α* = 0.05, *f* = 0.25, and power (1 – *β*) = 0.95, indicated that a minimum sample size of 24 participants would be required. All participants were selected from the Guangzhou Vocational University of Science and Technology, and they were all assessed using the Bergen Social Media Addiction Scale (BSMAS).

In accordance with a previous study (Luo et al., 2021), participants who scored 24 or higher on the BSMAS were classified as exhibiting a tendency toward SMA. In the current study, the inclusion criteria were as follows: a BSMAS score of 24 or higher, proficiency in Chinese, and normal or corrected-to-normal vision. Participants were excluded if they had any current mental health disorders, including but not limited to depression or anxiety, as well as other addictive behaviors such as substance abuse, gambling addiction, or gaming addiction. To enhance the reliability and validity of this study, an initial sample of 46 college students exhibiting characteristics of SMA was selected. During the study, two participants withdrew for unspecified reasons. Ultimately, a total of 44 college students completed the experiment, including 30 females and 14 males. Twenty-two were allocated to the control group and 22 to the training group.

### Procedure

This study was conducted in the laboratory of the School of Art and Media at Guangzhou Vocational University of Science and Technology. Each participant was required to complete behavioral experiments at Time 1, Time 2, Time 3, and Time 4. Each of these experimental sessions took about 15 min, and each involved participants completing a dot-probe task after providing informed consent. Additionally, the participants also complete the BSMAS questionnaire at each of the four designated time points.

### Measures

#### BSMAS

The BSMAS scale, originally developed by Andreassen et al. (2017), was used here in the version adapted for the Chinese context by Leung et al. (2020). This scale assesses the severity of an individual’s social media addiction over the preceding 12 months and comprises six Likert-type items, with higher scores indicating elevated levels of SMA. A sample item is, “I use social media to the extent that it negatively impacts my study and work.” All items are scored on a five-point Likert scale, from 1 = *very infrequently* to 5 = *very frequently*. Although the BSMAS references a 12-month timeframe, the prior validation and intervention study indicates that it is sensitive to short-term changes in social media use and related symptoms (Bottaro et al., 2025; Hou et al., 2019), as individuals tend to weight recent experiences more heavily when completing retrospective reports (Tov, 2012). Therefore, this study adopted the BSMAS as a standardized assessment tool to systematically investigate the changes in the severity of SMA among addicts before and after the intervention.

#### ABM training

The formal experiment used a set of 20 social media-related words and 20 neutral words as stimuli. These two types of materials were matched, respectively, in terms of similarity, frequency, and length, as detailed in our previous study (Zhao et al., 2022b). Following the procedure of MacLeod and Mathews (2012), the standard ABM procedure used in this study comprises three distinct stages: pre-test, training, and post-test. To verify the effectiveness of the ABM intervention, the intervention effects were tracked and analyzed at four time points: prior to training (Time 1), immediately following training (Time 2), 2 weeks post-training (Time 3), and 4 weeks post-training (Time 4).

#### Evaluation tasks

**Figure 1:**
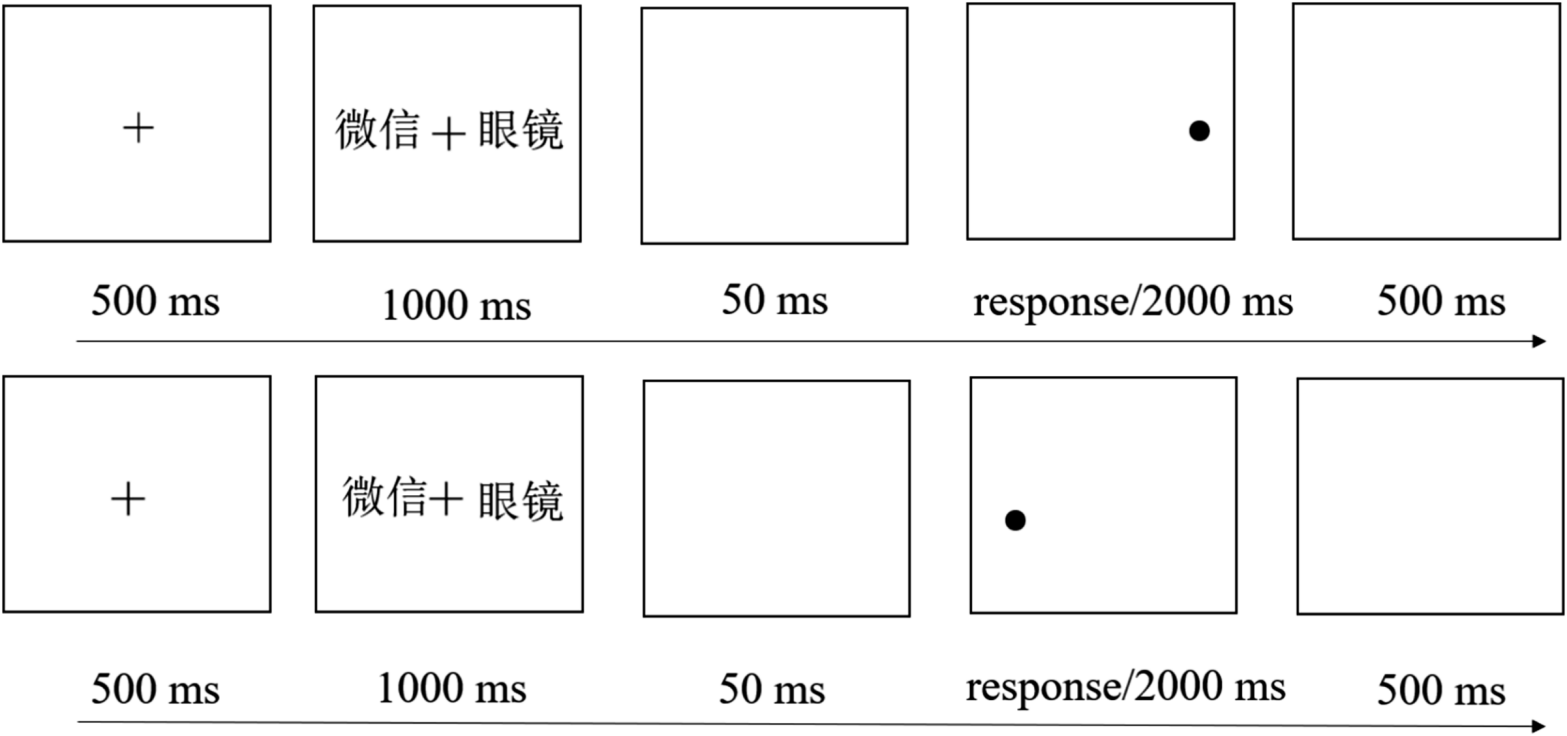
The timeline of evaluation tasks. 微信 = WeChat, 眼镜 = Glasses.

The evaluation tasks administered to the participants at the four time points were largely the same. Each task in the formal experiment comprised 240 trials. At the onset of each trial, a fixation cross (+) was centrally presented for 500 milliseconds (ms). Subsequently, a pair of words, one related to social media and one neutral, was presented bilaterally for 1,000 ms, with each word at an equal distance (40 mm) from the center of the screen. A probe dot was then randomly presented at the locations corresponding to one of the two words. Each word pair appeared four times, with each presentation corresponding to a random combination of picture location (left, right) and probe replacement (left, right). The dot probe had an equal probability (50%) of appearing at the location of either the word related to social media or the neutral word. A 50-millisecond interval then elapsed, and then the participants were instructed to press the F key if the probe dot had appeared on the left side and the J key if it appeared on the right side, as quickly and accurately as possible. The probe dot disappeared immediately following the participant’s response, with a maximum display duration of 2000 ms on the screen. The experimental procedure is presented in Figure 1.

#### Training phase

To minimize practice effects, some of the stimuli used during the evaluation tasks were replaced with new stimulus words in the training phase; otherwise, the experimental procedure was the same as the one used in the evaluation tasks. The training group participated in ABM training aimed at efficiently shifting the participants’ attentional resources away from cues related to social media. For the training group, the dot probe consistently appeared at the position of the neutral words (i.e., 100% contingency), whereas the control group received unbiased attention training, with the dot probe appearing at the positions of either the social media-related words or the neutral words with equal probability (i.e., 50% contingency). Following the completion of the pre-test, all participants began the intervention on the second day. Each training task comprised 240 trials and lasted for 15 min. Training was conducted once daily over seven consecutive days, totaling 1,680 correction training trials.

### Data analysis

Results were considered statistically significant when *p* was less than 0.05. Descriptive statistics, Chi-square tests, and t-tests were used to analyze the demographic characteristics of the participants. The AB scores were computed by subtracting the mean reaction time (RT) for dot probes at the location of social media-related cues (congruent condition) from the mean RT for dot probes at the location of neutral stimuli (incongruent condition). A positive score indicated AB toward social media relevant stimuli, a score of zero indicated no AB, and a negative score indicated attentional avoidance of social media relevant stimuli.

To assess the intervention effects of ABM training on AB toward social media relevant stimuli and SMA severity, a series of 2 (Group: training versus control) × 4 (Time: Time 1, Time 2, Time 3, Time 4) repeated-measures ANOVAs were carried out. A significant interaction effect was considered to warrant further examination of the temporal changes in the AB and SMA severity among participants in both groups.

## Results

### Demographics

There were no statistically significant differences in BSMAS scores, age, or gender distribution between the two groups. The demographic details are presented in Table 1.

**Table 1:** Comparison of demographic characteristics between the two groups (M ± SD).

The timeline of the evaluation tasks	Training group (*n* = 22)	Control group (*n* = 22)	*t*/*χ*2	*p*
BSMAS	25.05 ± 1.29	24.50 ± 0.96	1.59	0.12
Age	20.00 ± 1.23	20.36 ± 1.29	0.95	0.35
Gender (female/male)	16/6	14/8	0.42	0.52

### Attentional bias toward social media relevant stimuli

Data Screening: Using the same procedure as in our previous study (Zhao et al., 2022a), trials with incorrect responses, with RTs greater than 1,200 ms or less than 200 ms, or with RTs exceeding three standard deviations from the mean were excluded from further analysis.

**Figure 2:**
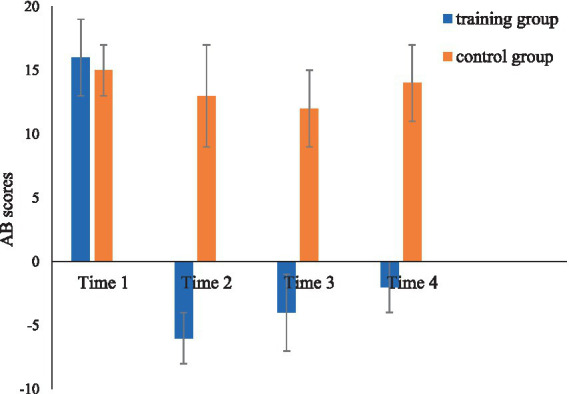
Changes in AB scores in the training group and the control group.

Results from ANOVA with group and time as independent variables and AB scores as the dependent variable revealed a significant main effect for groups [*F*(1, 42) = 26.43, *p* < 0.001, $\eta_{p}^{2}\;$ = 0.39]. Specifically, the training group exhibited a significantly lower AB score for social media-related information compared to the control group. The main effect of time was also significant [*F*(3,126) = 9.34, *p* < 0.001, $\eta_{p}^{2}\;$ = 0.18], as was the effect of interaction between group and time [*F*(3,126) = 5.84, *p* = 0.001, $\eta_{p}^{2}\;$ = 0.12]. Further analysis revealed no significant difference between the training group and the control group in AB scores at Time 1. The AB scores of the training group at Time 2, Time 3, and Time 4 were significantly lower than those at Time 1 [*t*(21) = 6.46, *p* < 0.001; *t*(21) = 4.29, *p* < 0.001; *t*(21) = 4.54, *p* < 0.001]. In contrast, no significant differences were found in the AB scores of the control group among Time 1, Time 2, Time 3, and Time 4 [*t*(21) = 0.67, *p* = 0.51; *t*(21) = 1.14, *p* = 0.27; *t*(21) = 0.36, *p* = 0.72]. A detailed comparison of the AB scores for both groups across different time points is provided in Figure 2.

**Figure 3:**
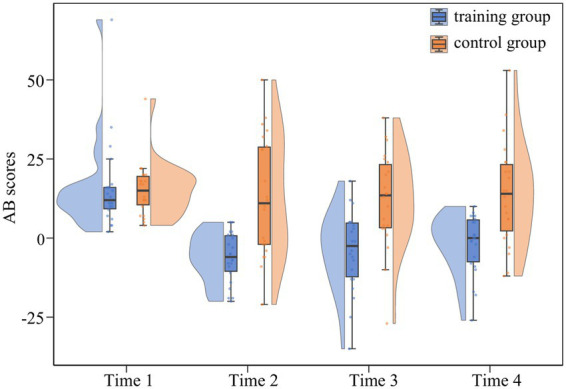
Raincloud plots of AB scores across four assessment time points in both groups.

Raincloud plots depicting the full distribution and individual data points are presented in Figure 3.

### BSMAS

**Figure 4:**
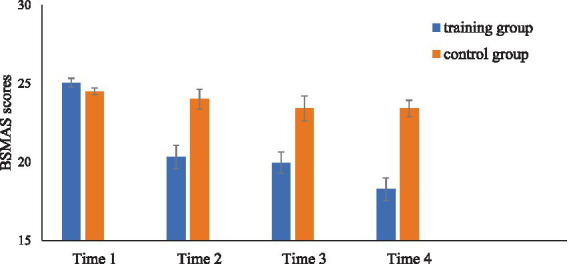
Changes in BSMAS scores in the training group and the control group.

The ANOVA results, with group and time as independent variables and BSMAS scores as the dependent variable, revealed a significant main effect of group [*F*(1, 42) = 20.11. *p* < 0.001 $,\eta_{p}^{2}\;$ = 0.32]. The mean BSMAS score of the training group was significantly lower than that of the control group. The main effect of time was also found to be statistically significant [*F*(3,126) = 27.73, *p* < 0.001, $\eta_{p}^{2}\;$ = 0.40]. A significant interaction effect was detected between group and time [*F*(3, 126) = 14.29, *p* < 0.001, $\eta_{p}^{2}\;$ = 0.25]. Further analysis demonstrated that the BSMAS scores of the training group at Time 2, Time 3, and Time 4 were all significantly lower than those at Time 1 [*t*(21) = 6.81, *p* < 0.001; *t*(21) = 7.70, *p* < 0.001; *t*(21) = 9.44, *p* < 0.001]. In contrast, the BSMAS scores of the control group at Time 1 were not significantly different from the later measurements taken at Time 2, Time 3, and Time 4 [*t*(21) = 0.81, *p* = 0.43; *t*(21) = 1.39, *p* = 0.18; *t*(21) = 1.84, *p* = 0.08]. A detailed comparison of BSMAS scores across different time points for both groups is provided in Figure 4.

**Figure 5:**
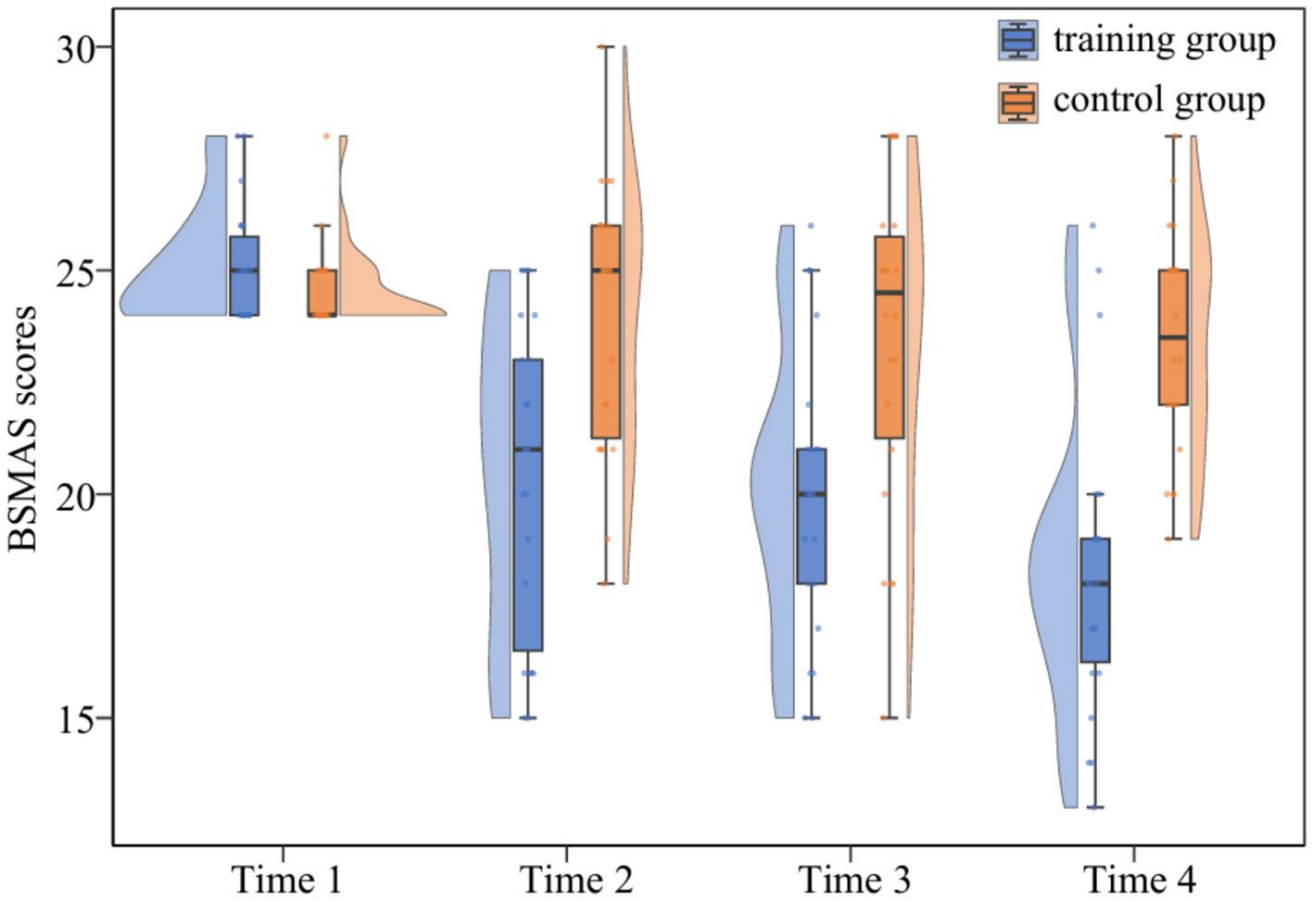
Raincloud plots of BSMAS scores across four assessment time points in both groups.

Raincloud plots depicting the full distribution and individual data points are presented in Figure 5.

## Discussion

Although multiple factors contribute to SMA, a substantial body of research has indicated a crucial role for cognitive factors, in the onset and persistence of addictive behaviors, the foremost of which is AB related to addiction information (Griffiths and Kuss, 2017; Nikolaidou et al., 2019; Zhao et al., 2022a,b). In the research field of substance addiction and behavioral addiction, ABM training, as a potential intervention approach, has been systematically explored by a large number of empirical studies (Robinson et al., 2022; Smith et al., 2020; White and Pohl, 2022). Typically, the effectiveness of such training is assessed based on its ability to reduce AB toward addiction-related stimuli and its ability to alleviate clinically relevant addiction-related symptoms. As a relatively novel form of addiction, SMA has predominantly been addressed through cognitive behavioral therapy interventions (Hou et al., 2019). To date, there is no existing research on the application of ABM training as an intervention for SMA. The present study therefore enriches current understanding of ABM training for SMA and provides a new empirical basis for the development of effective interventions for SMA.

Its results show that after ABM training, individuals with a tendency toward SMA exhibited changes in AB toward social media relevant stimuli, as well as reduced SMA severity, and the training effects remained significant over 4 weeks post-training. Previous intervention studies on SMA have mostly been conducted in a group format, with typical paradigms including structured group interventions based on cognitive behavioral therapy (Brevers and Turel, 2019; Chen et al., 2022; Dogan et al., 2019; Hou et al., 2019). This study, however, shifts its focus to the individual cognitive processing level, concentrating on the specific cognitive biases exhibited by SMA individuals. This study thus expands the scope of research on interventions and treatments for SMA, representing the first empirical evidence of the long-term efficacy of ABM training within the context of SMA research.

### Effect of ABM training on AB toward social media relevant cues

In this study, the training group and the control group each comprised 22 college students exhibiting addiction to social media. ABM training was used to correct the training group’s AB toward social media-related cues. At Time 1, the pre-test stage, there was no significant difference in the two groups’ AB scores toward social media-related stimuli. After seven consecutive days of ABM training, the AB scores of the training group changed significantly, showing a reversal in direction at Time 2, from an AB toward social media relevant stimuli to attentional avoidance. In contrast, the control group exhibited no statistically significant changes in AB scores, suggesting that following unbiased training, the group continued to display an AB toward social media relevant information. To evaluate the long-term effects of this intervention method, follow-up assessments were performed on both groups of participants at Time 3 and Time 4. The findings show that, although the AB scores of the training group exhibited an upward trend, they remained significantly lower than those recorded at Time 1. In contrast, the AB scores of the control group were consistently positive across all four time points and did not differ significantly from those observed at Time 1. Moreover, the corrective effects are sustained for up to 4 weeks post-training. This result agrees with prior research regarding the corrective effects of ABM training on addictive behaviors (Smith et al., 2020) and highlights the applicability of ABM training that targets addiction cues in the context of SMA. These findings provide clear support for Hypothesis 1, demonstrating that ABM training effectively reduces AB toward social media relevant cues in individuals with SMA.

Studies have shown that a key feature of addictive behaviors is the preferential allocation of attentional resources toward addiction-related cues when individuals with addiction are exposed to these stimuli (Bollen et al., 2021; Robinson, 1993; Robinson and Berridge, 2002; Smith et al., 2020; Zhao et al., 2022b). AB toward addiction-related information plays a critical role in the initiation, maintenance, and exacerbation of addictive behaviors (Bollen et al., 2021; Nikolaidou et al., 2019; Smith et al., 2020; Zhao et al., 2022b). The dual-process model posits that decision-making is characterized by the interaction between the impulsive system and the reflective system (Wiers et al., 2007; Wiers and Stacy, 2006). The emergence and persistence of addictive behaviors can thus be explained by an imbalance between these two systems: inadequate engagement of the reflective system results in reduced inhibitory control and working memory capacity, while overactivation of the impulsive system triggers cravings and AB toward addiction-related cues (Bechara, 2005; Wiers et al., 2007). In addition, according to the incentive sensitization theory (Robinson, 1993), the dopamine system becomes sensitized by repeated exposure to addictive substances, thereby amplifying the motivational salience of addiction-related stimuli and rendering these cues more prominent, ultimately inducing AB toward addiction-related information in individuals with addiction. Considering the plasticity of AB, several studies have demonstrated that ABM training that targets addiction cues exerts a positive influence in terms of preventing and treating addictive behaviors (Field and Eastwood, 2005; Schoenmakers et al., 2010; Smith et al., 2020). Individuals with SMA frequently exhibit excessive AB toward social media relevant stimuli (e.g., tweets, photos, videos) while neglecting important tasks and interpersonal relationships in real life. This study revealed that repeated ABM training for individuals with SMA tendency gradually redirected their attention from social media-related cues to non-social media-related cues, thereby diminishing their AB toward social media relevant stimuli. This shift can be interpreted as being indicative of changes at the cognitive levels, reflecting adjustments in individuals’ cognitive processing of social media relevant stimuli. Furthermore, ABM training that targets social media relevant cues may help individuals with addiction to better regulate their impulses toward social media use and thus mitigate their AB.

### Effect of ABM training on the social media addiction severity

Studies to date on the effectiveness of ABM training in reducing addiction symptoms have yielded inconsistent results. Robinson et al. (2022) demonstrated that ABM training was ineffective in reducing smokers’ cigarette cravings and ultimately failed to significantly improve smoking behavior. In contrast, the findings of the present study indicated that seven consecutive days of ABM training produced significant reductions in the severity of SMA. A meta-analysis of the effectiveness of ABM training and found that of the 18 studies included, 10 reported significant reductions in symptoms related to substance addiction (Heitmann et al., 2018). These positive effects were predominantly observed following multiple treatment sessions, indicating that the clinical benefits of this training method are more likely to emerge after repeated interventions. Zhao et al. (2022b) demonstrated a positive association between the SMA severity and AB toward social media relevant cues. Given the established positive correlation between AB toward social media relevant information and addiction severity, seven consecutive days of ABM training for individuals with SMA may potentially reduce addiction severity.

The present study evaluated the severity of individuals’ SMA using a self-report questionnaire. To investigate whether ABM training influences the severity of SMA in both the short term and the long term, assessments of SMA were conducted at four distinct time points: Time 1, Time 2, Time 3, and Time 4. The training group and the control group exhibited no significant differences in their BSMAS scores at Time 1, confirming homogeneity in initial SMA severity between the two groups. The training group’s SMA severity at Time 2, Time 3, and Time 4 were significantly lower than those at Time 1, whereas no significant differences were observed in the severity of SMA at Time 1, Time 2, Time 3, and Time 4 for the control group after unbiased training. These findings suggest that ABM training significantly decreased the SMA severity in the training group, thereby supporting previous research indicating that ABM training can alleviate addiction-related symptoms (Heitmann et al., 2017, 2021; Smith et al., 2020). These findings provide direct support for Hypothesis 2, which predicted that ABM training would reduce the severity of SMA in the training group. Cost-effective intervention training can thus assist individuals in reducing SMA, potentially enhancing and improving their mental health.

The incentive-sensitization theory posits that continuous exposure to intensely pleasurable stimuli, such as social media, can result in the hypersensitivity of specific brain reward systems, leading these systems to direct attention toward addiction-related cues (Seo and Ray, 2019). During ABM training targeting social media-related cues, individuals with a tendency toward SMA implicitly learn, through a series of structured exercises, to redirect their attentional resources away from social media-related information. This process gradually modifies their patterns of attention toward social media, fostering healthier attention allocation and reducing their reliance on social media. Consequently, this shift enables them to focus more on beneficial real-life activities, such as interpersonal interactions, hobbies, and personal development. Ultimately, this alteration diminishes the severity of SMA.

### Implications

The significance of researching ABM training as a possible intervention for SMA lies in its potential to provide a novel and effective intervention strategy. Traditional interventions, such as cognitive-behavioral therapy and self-help techniques, have shown limited success in treating SMA. ABM training offers a new and unique approach by targeting AB, which are believed to play a critical role in the development and maintenance of addictive behaviors. The results of this study show that ABM training can modify AB toward addiction-related cues and reduce the severity of SMA, with the modification effect persisting for at least 4 weeks. Thus, this method holds promise for the successful prevention and mitigation of SMA. As a new approach targeting cognitive mechanisms underlying SMA, this study provides an important empirical contribution to the development of effective prevention and intervention strategies for SMA.

The present findings demonstrated relatively strong effects of ABM on both AB and SMA severity. This pattern may differ from some previous studies in substance addiction for several reasons. First, social media relevant cues are highly prevalent and personally relevant in daily life, which may enhance the malleability of AB (Nikolaidou et al., 2019). Second, the targeted ABM procedure was specifically tailored to social media relevant cues, rather than general or substance-related cues. It was consistent with prior research suggesting that stimulus specificity improves ABM efficacy (Smith et al., 2020). Third, the sample consisted of young adults in a critical developmental period for behavioral regulation, and younger participants benefited more from ABM on bias change scores (Jones and Sharpe, 2017). These factors may collectively contribute to the relatively robust effects observed in the present study compared with the broader addiction literature.

## Limitations and future directions

Although ABM training has been extensively studied in addiction research, several issues remain to be explored. First, the long-term effects of ABM intervention on attentional bias and addiction symptoms remain unclear. Prior studies have predominantly focused on comparing pre-test and post-test differences, but there have been few longitudinal tracking studies (Robinson et al., 2022; Smith et al., 2020; White and Pohl, 2022). Although this study incorporated a tracking component, the follow-up period was limited to 4 weeks post-intervention due to potential participant dropouts and other constraints. Consequently, the duration of the positive effects of ABM training remains unknown. Future research could use longer-term longitudinal studies to assess the persistence of ABM training effectiveness.

Second, the number and duration of training sessions may also influence the durability of the effects. Prior studies have demonstrated considerable heterogeneity in the dosage and duration of attentional bias modification (ABM) interventions, with the number of training sessions ranging from 3 to 8 and intervention periods spanning 1–5 weeks (Fodor et al., 2020; Heitmann et al., 2018; Parvaz et al., 2021; Robinson et al., 2022). Future studies should systematically determine the optimal number of training sessions and duration to maximize the effectiveness of ABM training.

Third, the proportion of probe stimuli appearing in the locations of addiction-related versus neutral stimuli represents another area of inconsistency in existing studies. In most studies, probe stimuli consistently appear following neutral stimuli (Robinson et al., 2022; Smith et al., 2020); however, some studies have employed ratios of 80:20 or 90:10 (Kruijt et al., 2019; Tiggemann and Kemps, 2020). Future studies should systematically address this issue to more effectively correct attentional biases and mitigate addictive behaviors.

Fourth, the selection of stimulus materials may also influence the intervention effects. This study only used textual materials in the dot-probe task due to the diverse types of social media platforms used in China and the varying social media use preferences of university students across different disciplines. Identifying images that can uniformly capture the interest and attention of all participants is thus challenging. However, images are more visually intuitive, as evidenced by the study by McManus et al. (1996), whose research on eating disorders demonstrated that visual stimuli may be more effective in inducing AB compared to textual stimuli. Future studies should consider using images as stimulus materials to further explore the effectiveness of ABM training.

Fifth, expectancy effects may represent a potential limitation of the present study. Participants were informed about the general purpose and potential implications of the research, which may have enhanced outcome expectancies. However, both the ABM training group and the control group received identical information regarding the study rationale and procedure, thereby minimizing systematic group differences in expectancy. Thus, group differences in AB scores and SMA severity are unlikely to be fully explained by expectancy effects alone. Nevertheless, future studies could explicitly assess participants’ expectancies regarding intervention effectiveness to further isolate specific ABM effects from non-specific expectancy influences.

Finally, regarding experimental tasks, this study predominantly used the dot-probe paradigm to assess the intervention effects of ABM. It is worth investigating whether the training effects of ABM can be generalized to other AB measurement tasks, such as Stroop tasks.

## Data Availability

The original contributions presented in the study are included in the article/supplementary material, further inquiries can be directed to the corresponding author/s.
